# Realistic Numerical and Analytical Modeling of Light Scattering in Brain Tissue for Optogenetic Applications^1^,^2^,^3^

**DOI:** 10.1523/ENEURO.0059-15.2015

**Published:** 2016-02-02

**Authors:** Guy Yona, Nizan Meitav, Itamar Kahn, Shy Shoham

**Affiliations:** 1 Autonomous Systems Program (TASP); 2 Faculty of Biomedical Engineering; 3 Rappaport Faculty of Medicine and Institute, Technion - Israel Institute of Technology, Haifa, 31096, Israel

**Keywords:** beam-spread function, light scattering, Monte Carlo, Optogenetics

## Abstract

In recent years, optogenetics has become a central tool in neuroscience research. Estimating the transmission of visible light through brain tissue is of crucial importance for controlling the activation levels of neurons in different depths, designing optical systems, and avoiding lesions from excessive power density. The Kubelka–Munk model and Monte Carlo simulations have previously been used to model light propagation through rodents' brain tissue, however, these prior attempts suffer from fundamental shortcomings. Here, we introduce and study two modified approaches for modeling the distributions of light emanating from a multimode fiber and scattering through tissue, using both realistic numerical Monte Carlo simulations and an analytical approach based on the beam-spread function approach. We demonstrate a good agreement of the new methods' predictions both with recently published data, and with new measurements in mouse brain cortical slices, where our results yield a new cortical scattering length estimate of ∼47 *µ*m at λ = 473 nm, significantly shorter than ordinarily assumed in optogenetic applications.

## Significance Statement

For optogenetic stimulation to become highly controlled, reproducible, and safe, a thorough understanding of the deep-tissue scattered-light distributions that mediate the excitation is required. However, effective computation tools validated by actual measurements in brain tissue are currently still lacking. In this paper, we introduce, study, and validate new numerical and analytical approaches for modeling the distributions of light propagating through brain tissue. We show that both methods lead to consistent results and use the much faster analytical method to iteratively extract the optical parameters from new measurements, suggesting that light penetration into cortical tissue is significantly less than usually assumed. The new level of faithfulness could assist in designing experimental setups and optical interfaces, and help interpret optogenetic experiments.

## Introduction

Optogenetic neuromodulation is playing an increasingly central role in neuroscience research and emerging applications (Deisseroth, 2011), with major efforts being directed toward the discovery and development of advanced optogenetic probes (Yizhar et al., 2011; Zhang et al., 2011) and related miniature devices (Deisseroth and Schnitzer, 2013). However, relatively little attention has been given to elucidating and characterizing the passage of light in brain tissue at the relevant visible wavelengths and illumination geometries: because light-tissue interactions at these wavelengths is strongly scattering-dominated, light transfer is heavily affected by multiple scattering events resulting in complex light distributions where the photons deviate considerably from their original directions. Estimating the resulting light distribution is of crucial importance for multiple aspects of optogenetic research including the control and analysis of neuronal excitation levels, comparison of the relative merits for different applications of probes with different excitation spectra, the design of effective optical systems for delivering sufficient light power density to target regions (Pashaie et al., 2014), and for avoiding lesions and light toxicity (Frigault et al., 2009) that may result from excessive light absorption.

Despite this central importance in interpreting and designing optogenetic experiments, methodical treatment of tissue light transport in this context has been sparse. To date, the central approaches used for studying relevant scattered light distributions in rodent brains were based on a Kubelka–Munk (KM) model fit to empirical results (Aravanis et al., 2007; Adamantidis et al., 2007; Yizhar et al., 2011; Foutz et al., 2012), and on Monte Carlo (MC) simulations of light transport (Bernstein et al., 2008; Chow et al., 2010; Kahn et al., 2011). Unfortunately, the generality and applicability of each of these approaches suffers from major limitations. The KM model (Kubelka, 1948) is a one-dimensional model describing the propagation of light through a diffuse scattering medium (with no absorption), based on two coupled differential equations that describe the change in the intensity at each point in the slab based on the change of two fluxes: the transmitted light flux and the backscattered flux. However, the KM model is based on fundamental assumptions that are inconsistent with light scattering in tissue geometries and length scales relevant to optogenetics: (1) it assumes isotropic scattering, which becomes true only at the diffusive regime, depths of multiple millimeters, whereas at the distance scales and wavelengths relevant to optogenetics, scattering is highly anisotropic; and (2) it assumes isotropic illumination (i.e., illumination from an infinite uniform plane), thus neglecting the finite geometry and size of the illumination, which is typically of comparable size to the tissue of interest. The various limitations of the KM model, as suitable for capturing light scattering, are extensively discussed by Neuman and Edström (2010). Likewise, the published MC calculations (Bernstein et al., 2008; Chow et al., 2010; Kahn et al., 2011) were based on scattering parameters for human brains (Yaroslavsky et al., 2002), and are unvalidated by empirical data (see Discussion). More recently, more extensive sets of measurements for estimating the optical parameters using different experimental strategies were performed in several subcortical adult mouse brain areas (Al-Juboori et al., 2013) and in adult rat brains (Azimipour et al., 2014). Although important, broad empirical measurement datasets do not provide the type of generality required for tackling a diversity of practical cases and designs, and leaves open the need for a complementary quantitative, practical, and empirically validated modeling framework for optogenetic light propagation.

In this work, we apply two independent methods for detailed modeling of the transmission of light launched from a monochromatic optical fiber light source across various thicknesses of brain tissue: the MC method (Fang, 2010) and the beam spread function (BSF) method (McLean et al., 1998), which is applied in this context for the first time. MC simulation uses repeated numerical random sampling, whereas BSF is based on convolutions of analytical beam propagation Green's function (an impulse response, which is used via the superposition principle to obtain a solution to more complex initial or boundary conditions). Despite the very different methodologies behind these two methods, we show that their results are both mutually consistent and in agreement with both published and new empirical brain-scattering results. Finally, we discuss the new approaches’ relative merits and limitations, as well as directions for future study.

## Methods

### General considerations

In our models and in previous experiments (Aravanis et al., 2007; Yizhar et al., 2011), an optical fiber was located adjacent and perpendicular to an ex-vivo slice of mouse brain tissue. The fiber was emitting light in wavelength λ= 473 nm, its core radius was 100 μm and numerical aperture (NA) of 0.37. These parameters were also used by Aravanis et al. (2007). We assume that the spatial and angular intensity distributions of the emitted light at the fiber surface are uniform and constant. This assumption holds because the electric field distribution at the fiber tip is composed of superposition of all the linearly polarized modes (amplitude distributions that remain unchanged during propagation in a fiber) supported by the fiber, which are very numerous. The number of modes (*M*) is typically estimated from the fiber's normalized frequency $V=2\pi \left(\frac{r_{core}}{\lambda }\right)NA=491.5$; (Okamoto, 2006), which indeed implies a very large number of modes $M\approx \frac{4V^{2}}{\pi^{2}}\approx 10^{5}$.

Another important parameter is the beam divergence at the fiber tip, because it determines the angular distribution of the light emanating from each point at the fiber tip. If we assume that the fiber is touching the tissue (i.e., no other interface between them), the half-angle of divergence θ_div_ is

$$\theta_{div}=\sin^{-1}\left(\frac{NA}{n_{tissue}}\right)=15.8{}^{\circ},$$

where $n_{tissue}=1.36$ is the refractive index of the brain tissue (Vo-Dinh, 2003). We assume a homogenous and isotropic (i.e., there is no preferable direction in the tissue) scattering tissue, characterized by a scattering coefficient *µ_s_* (cm^−1^), a weak absorption coefficient *µ_a_* (cm^−1^, *µ_a_* ≪ *µ_s_*), and a high anisotropy coefficient *g* (dimensionless) which represents a tendency for strongly forward scattering at each scattering event. Using the two methods described below, we calculate local intensity (the radiant power passing through a unit surface) in different points across the tissue.

### Monte Carlo simulation

The MC simulation is based on the Mesh-based Monte Carlo (MMC) code developed by Fang (2010), version 0.9.5. Because the MMC software is based on an infinitely narrow beam (pencil beam) light source, we adapted it to a more complex light source using a three-step process:

(a) The 3D pencil-beam response was produced by simulations performed at a logarithmic lateral resolution spanning from 1.2 µm near the central beam to 47 µm at the edge of the simulated volume, and an axial resolution of 5 µm, with 10^8^ simulated photons. The response was then resampled by a uniform isotropic grid of 5 µm.
(b) The 3D pencil-beam response was rotated in 64 intervals over the inclination angle $\theta_{div}$ along the rotation axis, which is the entrance point of the pencil beam into the tissue. Sequentially, the result was rotated over a full circle in 64 intervals along the azimuthal direction. The results of all the rotations were summed up to form the angular light pattern that was emitted from each point of the fiber tip (Fig. 2*a*). In effect, the rotations can be formulated in terms of angular convolution with unit vectors that span the inclination angles $\left[0,\theta_{div}\right]$ and the azimuth angles $\left[0,2\pi \right]$.
(c) To take into account the fiber-tip area, the result of the previous stage was convolved with a sampled disk of the same diameter as the fiber (Fig. 2*a*).

### Beam-spread function simulation

**Figure 1.**
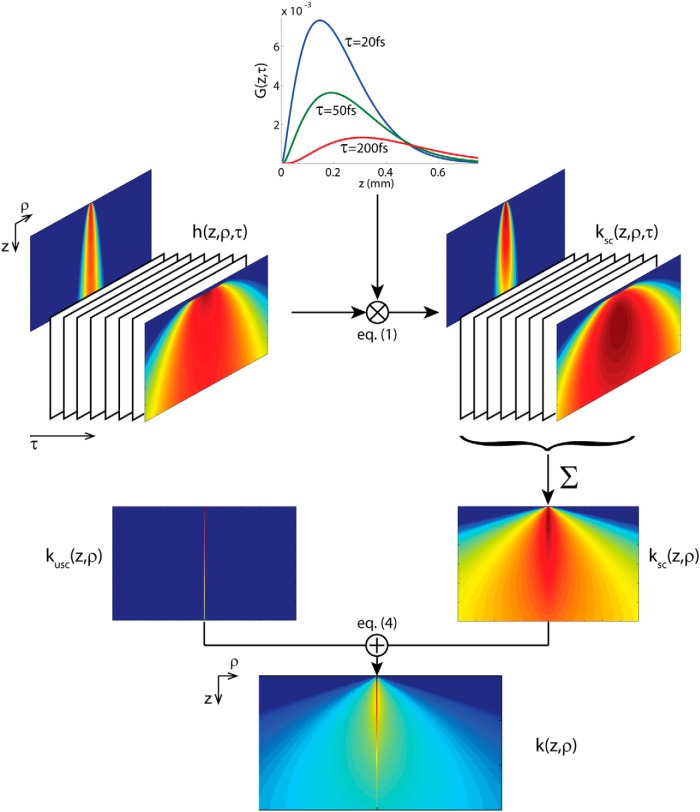
Graphical depiction of the BSF calculation process: the distribution of the scattered photons *k*_sc_(*z*,**ρ**,τ) is a product of the spatial distribution *h*(*z*,**ρ**,τ) and the temporal dispersion distribution *G*(*z*,τ) (Eq. 1). The time-dependent distribution is integrated over time to obtain the intensity values *k*_sc_(*z*,**ρ**), and added to the distribution of the unscattered photons *k*_usc_(*z*,**ρ**) for the total distribution *k*(*z*,**ρ**) (Eq. 4), called the BSF. Note that the equations also include absorption effects that were omitted from the figure for simplicity.

The BSF method (McLean et al., 1998) is a uniquely powerful solution for approximating light distributions in highly forward scattering media where the higher-order effects of photons coming via multiple paths of various lengths, thus resulting in time dispersion of the light intensity, is also incorporated. The method applies an analytical approximation for unidirectional pulsed source propagation in a turbid medium, which serves as a Green's function that can be used to solve more general problems (i.e., via angular and spatial convolution): the BSF $k\left(z,\rho ,\tau \right)$ is the intensity distribution of light from a pulsed source normalized to the pulse's energy, after propagating a distance z in the medium, where ρ is the radial position vector, and τ=t-z/c is the multipath time (c is the speed of light in the medium, t is the time since the photons start propagating, *τ* = 0 for unscattered photons). McLean et al. (1998) present a useful decomposition of the scattered photons BSF into a product of a normalized temporal dispersion distribution function $G\left(z,\tau \right)$ and a normalized spatial-angular distribution function $h\left(z,\rho ,\tau \right)$ (Fig. 1, top):

(1) $$k_{sc}\left(z,\rho ,\tau \right)=e^{-\mu_{a}\left(z+c\tau \right)}G\left(z,\tau \right)\cdot h\left(z,\rho ,\tau \right),$$

where *µ_a_* is the absorption coefficient. Following McLean et al. (1998), we use the time-dependent spatial distribution function (derived from the time-independent small-angle approximation):

(2) $$h\left(z,\rho ,\tau \right)=\frac{3}{4\pi \tau \;cz}\exp\left(-\frac{3}{4}\frac{\rho^{2}}{\tau \;cz}\right),$$

and the Gamma probability density function is used as the temporal distribution function (equivalent to a normal distribution when the variable, τ, is positive definite):

(3) $$G\left(z,\tau \right)=\frac{\mu }{\sigma^{2}\Gamma \left(\frac{\mu^{2}}{\sigma^{2}}\right)}{\left(\frac{\mu \tau }{\sigma^{2}}\right)}^{\frac{\mu^{2}}{\sigma^{2}}-1}\exp\left(-\frac{\mu \tau }{\sigma^{2}}\right),$$

where μ and σ are the first and second moments of *τ*, respectively, and are dependent only on the first and second moments of the cosine of the scattering angle θ (McLean et al., 1998 provides the formulas), and $\Gamma \left(x\right)$ is the gamma function. The BSF of the scattered photons, $k_{sc}\left(z,\rho ,\tau \right),$ was calculated for the tissue optical parameters (same as for the MC method) and integrated over time. The unscattered photons were added next to obtain the combined BSF (Fig. 1, bottom):

(4) $$k\left(z,\rho ,\tau \right)=\delta \left(\rho \right)\delta \left(\tau \right)e^{-\left(\mu_{a}+\mu_{s}\right)z}+\left(1-e^{-\mu_{s}z}\right)k_{sc}\left(z,\rho ,\tau \right).$$

**Figure 2.**
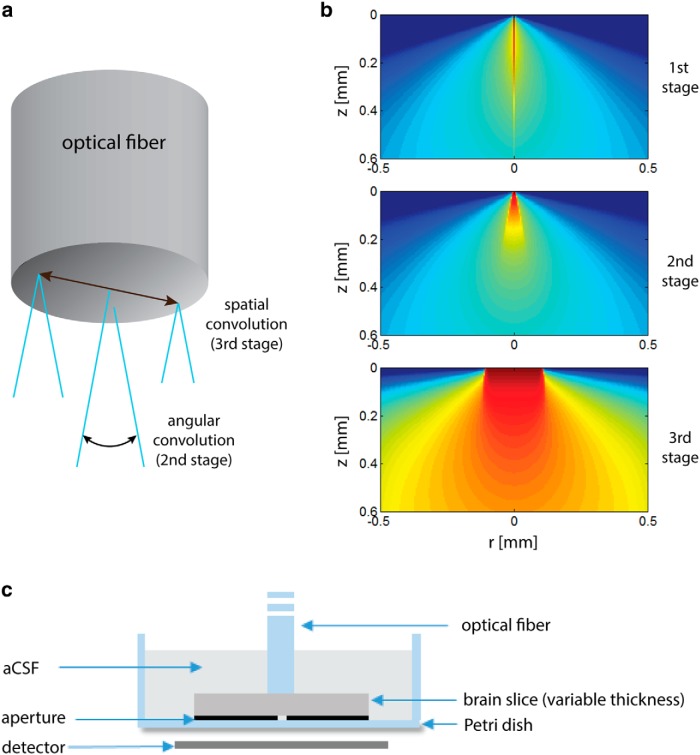
***a***, Simulation procedure: the 1st stage is the calculation of a 3D pencil-beam response, the 2nd stage is the angular convolution of the pencil beam response, and the 3rd stage is a spatial convolution with the fiber tip area. ***b***, Simulation outcomes of the various simulation steps. For better visualization, all of the figures are in log scale. ***c***, Illustration of the experimental setup cross-section.

Subsequently, angular and spatial convolutions were performed to obtain the propagation of light from the fiber’s tip, as was done with the MC method (only Steps 2 and 3 are required here, because the Green's function is calculated for the entire volume; Fig. 2).

The calculation of the BSF pencil beam is considerably faster than MC calculation with comparable accuracy (several seconds compared to hours). As a result, curve fitting can be more readily used to obtain the optical parameters of a sample. We implemented the accelerated gradient search method (Beck and Teboulle, 2009) to find the scattering coefficient and anisotropy factor of the published data manually extracted from Aravanis et al. (2007) and of our measurements (see Fig. 5).

### Experimental setup

All procedures were conducted in accordance with the national ethics committee for animal experimentation and with the approval of the Committee on Animal Care at The Technion-Israel Institute of Technology. Mouse brain slices were illuminated from above with a blue laser (473 nm) emanating from a fiber of 200 μm in diameter with NA of 0.37 (Thorlabs BFL37-200). The light intensity was measured through a small aperture (∼50 μm diameter) using a power meter (Newport 818-ST), when the fiber tip is adjacent to the slice surface, above the point of maximal intensity (Fig. 2*c*). Oblique, semi-coronal brain slices were obtained from two female mice (5 months old, C57/BL6 strain) from the same litter. The slice thicknesses used were 150, 200, 300, 400, 500, and 600 μm. Four measurements per slice were taken in neocortical areas, with two to three slices of each thickness obtained per animal (n=16–20 for each slice thickness). Because we found greater scattering near the white matter at the deep layers of the cortex, the measurements were performed at central locations. The slices and the fiber were submerged in artificial CSF throughout the measurements, and were oxygenated until shortly before the measurement. In addition, radial intensity profile was obtained for the 300- and 600-μm-thick slices, by moving the fiber tip laterally in 50 μm intervals to 1 mm. The measured results were normalized to the measured light intensity without the slice (at the same height above the aperture) divided by the theoretical predicted attenuation due to geometric beam spreading.

## Results

**Figure. 3.**
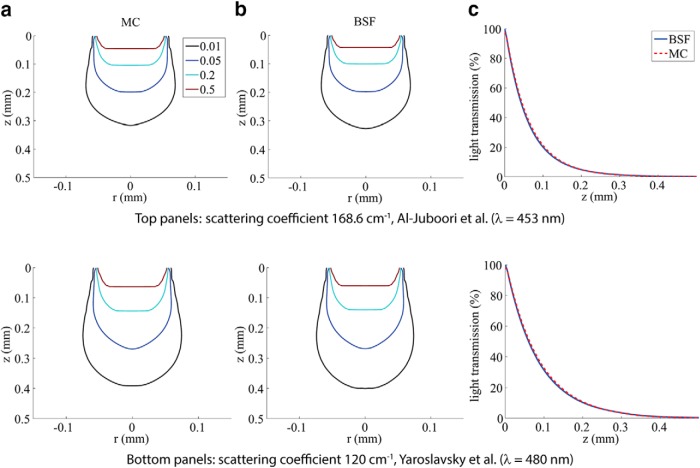
Comparisons of MC and BSF methods for two different tissue-scattering parameter settings: *µ_s_* =168.6 cm^−1^ (top; from Al-Juboori et al. 2013) and *µ_s_* =120 cm^−1^ (bottom; from Yaroslavsky et al. 2002). ***a***, Contour maps of the light distribution in the tissue, created using the MC method. ***b***, Contours created using the BSF method. The iso-intensity lines are at 50, 20, 5, and 1% of maximum. ***c***, Light transmission curves along the *z*-axis.

We first examined the accuracy of BSF method by comparing it to MC simulation results using two sets of previously published brain tissue optical parameters: scattering coefficient *µ_s_* =168.6 cm^−1^ from Al-Juboori et al. (2013) measured in the mouse’s pedunculopontine nucleus using 453 nm blue light and *µ_s_* =120 cm^−1^ from Yaroslavsky et al. (2002) measured in human brain gray-matter tissue using 480 nm blue light. Figure 3*a* shows contour maps of the calculated light transmission using the MC method, and Figure 3*b* shows the same using the BSF method. The fiber diameter used for the models in this figure is 100 μm and the NA is 0.22 (as reported by Al-Juboori et al., 2013). Due to the lack of better data, we used the absorption coefficient and anisotropy factor of native human gray matter from Yaroslavsky et al. (2002): *µ_a_*= 0.6 cm^−1^ and *g* = 0.88 in both cases. These results illustrate that the analytical BSF method generally follows the MC predictions quite closely for a parameter regime, which is relevant for optogenetics.

**Figure. 4.**
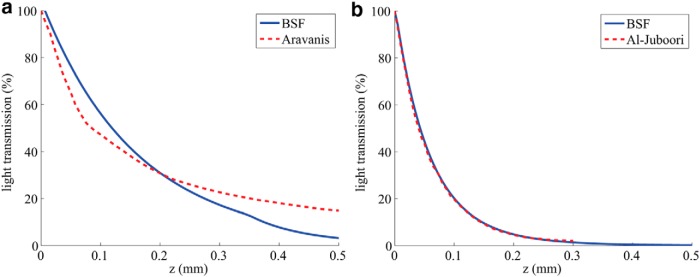
Transmission of light along the *z*-axis, comparing published experimental results (red dotted curves) with BSF method (blue curves). ***a***, Best-fit curves to measured data from Aravanis et al. (2007). ***b***, Experimental data from Al-Juboori et al. (2013) measured from the peduncopontine tegmental nucleus; the optical parameters used for the BSF curve are explained in the text.

Next, we compared the models’ predictions to two published experimental accounts on transmission along the *z*-axis, that is, the fiber’s central axis (Fig. 3; Aravanis et al., 2007; Al-Juboori et al., 2013). First, we compared the models’ results of Figure 3*c* to the published decay curves by Al-Juboori et al., (2013; Fig. 4*b*). As discussed above, the results of the MC and the BSF methods are in excellent agreement [root mean square (rms) transmission error of 0.52% over the range 0–1 mm], and we find that both provide a very good fit to the experimental measurements (rms error of 0.97% over the range 0–0.3 mm). In contrast, the model-based simulations are in poor agreement with the experimental measurements of Aravanis et al. (2007). The comparison used the experimental setup geometry of Aravanis et al. (2007), where the fiber tip remained in a fixed height while the tissue samples were being replaced, and no aperture was used. It can be shown that the light attenuation curve obtained this way is equivalent to depicting the integrated light intensities over the entire layers. A version of the BSF model was adapted to implement this integration, and best-fit curves to the data from Aravanis et al. (2007) used this modification. The obtained optical parameters were as follows: scattering coefficient 60.7 cm^−1^, absorption coefficient 0.62 cm^−1^, and anisotropy factor *g* = 0.89.

**Figure. 5.**
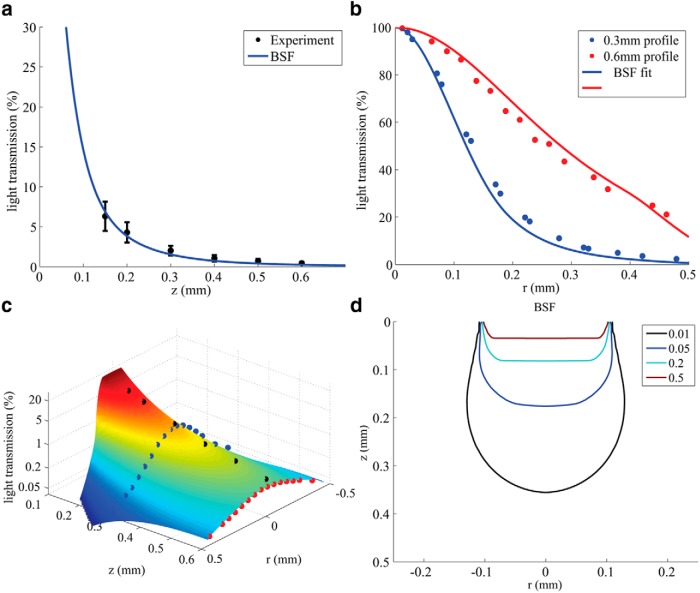
Experimental results: light transmission in a mouse cortex along the *z*-axis (***a***) and the radial axis (***b***) at 300 μm (blue) and 600 μm (red). The solid lines are the best fit of the BSF model. ***c***, Surface plot of the simulated light distribution in the slice, obtained with BSF model using the experimentally estimated parameters (*µ_s_* = 211 cm^−1^, *g* = 0.86), with overlaid experimental measurements (colors matched to ***a*** and ***b***). Light transmission is in log scale. ***d***, Contour map of the simulated light distribution.

Finally, we compared model-based predictions to the new set of measurements performed in adult mouse cortical slices (Fig. 5). To estimate the tissue’s scattering parameters, we searched for a BSF model that simultaneously provided the best fit to both the axial and lateral profiles (obtained parameters: scattering coefficient 211 cm^−1^ and anisotropy factor *g* = 0.86). The results again demonstrate an excellent agreement between the respective simulation methods, and between them and the experimental curves. Overall, these results exhibit a strong attenuation of the light intensity along the *z*-axis due to multiple scattering: the intensity is reduced to 50% at a depth <40 µm, and to just a few percentages at depths exceeding 200 µm (mean free path is 47 µm). This finding highlights how the gray matter’s high density leads to a large number of scattering events over a relatively very short distance.

## Discussion

In this study, we sought to develop and experimentally validate solutions for realistic modeling of optogenetic light delivery using an optical fiber embedded in brain tissue. MC simulations, a generally accepted numerical method for simulating light propagation in biological tissues (Zhu and Liu, 2013), have already been extensively applied to this problem, however, generally using inappropriate tissue parameters (Table 1) and without capturing the input light source's spatial and angular properties; these issues are particularly important in light of the general lack of experimental validation of simulation results. Additionally, we adapted and extended a powerful analytical method for estimating scattered light distributions, the BSF, and carefully compared it with the MC results and to experimental measurements. Importantly, the results of the analytical solutions were found to be highly consistent with the MC simulation results, with the published attenuation profile measurements by Al-Juboori et al. (2013) in mouse subcortical regions (Fig. 4*b*), and with a new set of cortical attenuation measurements (Fig. 5). The model-based iso-intensity contours in cortical slices (Fig. 5*d*) are also in rough agreement with the measured results found in Yizhar et al. (2011, their Fig. 3E), which is itself in stark disagreement with the attenuation graph of Aravanis et al. (2007; reproduced by Yizhar et al. 2011, their Fig. 3B). Our method for measuring and estimating the parameters uses a geometry and fits to localized measurements that are directly relevant to optogenetics and does not rely on the multiple assumptions behind diffuse reflectance estimates (Azimipour et al. 2014).

The very good inter-model agreement found is probably as good as one can obtain, given that there are several reasons to expect small discrepancies between the two methods. First, the BSF formulation uses several approximations and assumptions. For example, the temporal probability density is modeled as a Gamma distribution, although more recent work (Funk and Pfeilsticker, 1999; Theer and Denk, 2006) suggests the lognormal distribution may be a more accurate model. Moreover, the methods have some inherent numerical errors, especially as a result of integration, interpolations, and the limited volume (spatial, temporal, and directional) allocated for the Green function. Likewise, differences from the empirical measurements can be attributed to tissue inhomogeneity, approximations in the experimental setup (e.g., neglecting the polystyrene and air layers between the slice and the detector, errors in slice thickness, etc), and errors in the estimated optical parameters used. Finally, although the methods were applied here to a uniform light source, it can be easily accommodated to other beam profiles (e.g., Gaussian beam, multiple fibers, etc). It is, however, important to note that like almost all current methods that describe the propagation of light in a turbid media, both methods do not take the wave properties of the light into account; thus, when using a coherent source, the interference of photons with different phases (due to different optical path lengths) will form a speckle pattern that can also reduce the total illumination level at each position.

**Table 1.** Brain tissue optical parameters

**Source**	**Brain sample**	**Wavelength, nm**	**Scattering coefficient, cm^−1^**	**Anisotropy factor**
Our experiment	Adult mouse cortex, (5 months old)	473	211	0.86
Aravanis et al. (2007)	Mouse cortex	473	60.7	0.885
Al-Juboori et al. (2013)	Mouse subcortical, (6–8 weeks old)	453	168.6	—
Azimipour et al. (2014)	Rat cortex	532	∼170	∼0.9
Yaroslavsky et al. (2002)	Human gray matter	480	120	0.88

Using the new BSF method, the scattering coefficient and anisotropy factor were estimated in measurements performed in mouse cortical slices. This estimation procedure was based on simultaneously optimizing the mean squared error (MSE) fit to both the axial and two lateral curves (Fig. 5) using only two scattering parameters (or “degrees of freedom”). This iterative fit procedure was facilitated by the tremendous speed gain of the BSF method relative the MC numerical simulations (∼10 sec per BSF computation versus ∼10 hr per MC to achieve a comparable accuracy on a modern desktop PC).

Table 1 compares our results to estimates of these parameters based on the published data by Aravanis et al. (2007), as well as related parameter estimates obtained in the adult mouse pedunculopontine tegmental nucleus (Al-Juboori et al., 2013), in adult rat cortex (Fig. 5; Azimipour et al., 2014) and in human gray matter (Yaroslavsky et al., 2002). This comparison illustrates the much stronger scattering in mouse cortex seen in our results than indicated by Aravanis et al. (2007) and is generally more consistent with the stronger scattering observed by Al-Juboori et al. (2013) and Azimipour et al. (2014). Noting the considerable difference in the optical parameters between human and mouse brains, and between brain areas, we advise to refrain from using them interchangeably. Indeed, these findings explain the observations in Bernstein et al. (2008), who report a ratio of ∼0.73 in the diameters of the 1% and 10% contours of light attenuation between their measurements in mouse brain slices and MC simulations, which were based on human brain optical parameters. Note, however, that these estimates should be used with caution if scattering is expected to change. For example, measurements should be performed on tissue samples obtained from younger animals, as their scattering has been shown to be weaker (Oheim et al., 2001).

These results therefore put forward the BSF as a viable analytical approach toward a better understanding of light propagation and interactions in optogenetics and related fields, whereas use of the Kubelka–Munk model should be discouraged due to its theoretical inadequacy. As we have shown, the BSF can be used both for calculating the light distribution in brain tissue, and for estimating the optical parameters from measured attenuation curves. Because the BSF is an analytical method, it is considerably faster than a MC simulation, enabling its implementation to curve fitting algorithms that obtain the optical parameters of the tissue and require multiple calculations of the attenuation curve with different parameters. A major limitation of light propagation models in brain tissue (including the ones portrayed here) is the assumption of homogeneity, whereas the cortical layers have different cytological properties, and necessarily also different optical parameters. These can be obtained in a similar manner, by performing the attenuation curve fitting in parts using the histologic layers’ depths. A useful, immediate application of the BSF method for experiment design is to determine the tissue volume illuminated to various levels by different optical fibers (Table 2). It is noted that exact calculation of the light intensity inside the tissue is a prerequisite for determining the excitation from optogenetic stimulation, but does not completely describe the excitation from optogenetic light. To estimate the excitation properly, one has to know the cell morphology, the distribution of the light-sensitive channels and the baseline excitability from subthreshold oscillations. Moreover, *in vivo* optogenetic stimulation is also prone to absorption by blood (Azimipour et al., 2015), which is highly non-homogenous and remains untreated in our model.

**Table 2.** The illuminated tissue volumes for various commercial optical fibers

**Fiber properties**		**Volume illuminated (10^−3^× mm^3^), %**			
**NA**	**Diameter, µm**	**>50**	**>20**	**>5**	**>1**
0.1	25	0.02	0.04	0.09	0.20
0.22	50	0.06	0.14	0.34	0.79
0.22	105	0.29	0.68	1.48	3.28
0.22	200	1.07	2.56	5.53	13.7
0.22	365	3.62	8.71	19.5	61.7
0.37	200	1.05	2.52	5.52	14.3
0.39	200	1.05	2.51	5.50	14.4
0.39	300	2.39	5.75	12.7	38.1
0.39	400	4.30	10.4	23.3	79.4
0.48	400	4.76	10.3	23.1	79.0

Threshold is in percentage of maximal illumination.

A BSF solver is available at: niel.net.technion.ac.il/software.

## Synthesis

The decision was a result of the Reviewing Editor Zoltan Nusser and the peer reviewers coming together and discussing their recommendations until a consensus was reached. A fact-based synthesis statement explaining their decision and outlining what is needed to prepare a revision is listed below. The following reviewers agreed to reveal their identity: Thomas Oertner

Both reviewers agreed that the MS deals with a very important issue: light scattering in brain tissue. The authors applied combined theoretical, modelling and experimental approaches to predict the light intensities in the brain upon optic fiber illumination. The reviewers agreed that the topic is important, the approaches are generally valid and the results are interesting. There are, however, issues (some major!) that need to be addressed prior to publication. For details, please see the comments of the reviewers below.

1) For the intended application, estimating the number of neurons stimulated at a certain light intensity, it would be very useful to provide the volumes of tissue illuminated at 100-50%, 50-10%, 10-5%, and 5-1% of the max intensity, at least for the example shown in Fig. 4.

2) One of the few things the optogenetics user can freely chose is the diameter and NA of the multimode fiber. It would be nice to provide some guidance (e.g. table) how the fiber diameter affects the illuminated volume (see comment #1), keeping total power and wavelength constant.

3) Heating artifacts are a serious issue in in vivo optogenetics. I wonder if 99% of the injected power will be absorbed by the tissue within the 1% contour line, or is a significant fraction of photons re-entering the light fiber, being essentially 'reflected' by the scattering process? The Monte Carlo simulation should be able to provide an estimate of re-entry. Please quantify and discuss.

4) The phrase 'optogenetic light scattering' in the title is a bit odd, what about 'modeling of light scattering ... for optogenetic applications' instead.

1) The beam spread function with time dispersion method (BSF) should be described in more detail and more precisely.

2) Acute slices were "oxygenated until shortly before the measurements".

For how long were the slices measured?

Did the optical properties of the slices not change during measurements?

3) pages 12-13 - Why is the scattering coefficient in Figure 4, where the data is fitted to the experimental result, different from the scattering coefficient in Figure 3, where the results are compared with the data from other publications?

4) Is Fig 4c the same as the top panel in Fig 2b?

5) "new cortical scattering length estimate of ∼47µm"

Please give the wavelength of the measurement in the abstract, too. It would be useful to inform the readers right at the beginning.

6) "based on two coupled differential equations whose parameters are the scattering and absorption coefficient per unit thickness (which are not the same as the transport coefficients µa, µs)"

Please define the symbols for the scattering and absorption coefficients. Is "transport coefficient" the same as "transmission coefficient"?

7) "We assume that the spatial and angular intensity profiles of the emitted light from the fiber are uniform and constant."

Is this statement meant along the beam? Do you mean Gaussian or something similar, or a completely uniform beam intensity?

8) Numbering the formulas would improve in-text citations. Explaining all symbols in the formulas would also be useful. There is at least one missing symbol definition per formula.

9) "We assume that the scattering tissue is homogenous and isotropic (i.e. there is no preferable direction in the tissue)."

The authors have written a few sections earlier that "at the distance scales and wavelengths relevant to optogenetics, scattering is highly anisotropic". Please make your text consistent.

10) "As in many other models of light propagation in turbid media, the effects of coherence and polarization that result from the light wave properties are not considered."

How is this statement related to this work? Are these parameters also neglected?

11) According to Fig 1, it seems that back scattering was highly neglected or has 0 probability in the MC simulation. Please explain in more detail, or, alternatively, please indicate this approximation in the text.

12) The outermost cortical layer may be different from the inner cortical layers and as light should always penetrate through the surface, this highly determines the final penetration depth of the beam, thus the achievable depth resolution of i.e. microscopy.

13) page 3 - "KM model is based on fundamental assumption that are inconsistent with light scattering"

This inconsistency should be explained in more detail.

14) page 3 - "The published MC calculation were based on very different scattering parameter of human brains"

The term "very" should be clarified.

15) pages 3 and 4 - On page 3 the authors state that "generality and applicability" of MC simulation and the KM model "suffers from major limitation". Still, on page 4 they start to use MC as one of the proposed methods.

What is the novelty in the proposed new MC method? It would be useful to clarify the difference further.

16) page 10 - Why were different optical fibers used in modelling and in experiments (NA=0.22 versus NA = 0.37)

17) page 13 - The experimental and simulated data are compared only in Figure 4, and only absorption is compared, and only with the BSF result. It would be useful to show a contour map of the real experimental data to be able to more clearly demonstrate the applicability of the method.

18) page 17 - In Table 1 it would also be useful to show the age of the animal when it is available.
